# A Lower Sodium Neapolitan Pizza Prepared with Seawater in Place of Salt: Nutritional Properties, Sensory Characteristics, and Metabolic Effects

**DOI:** 10.3390/nu12113533

**Published:** 2020-11-17

**Authors:** Paola Iaccarino Idelson, Ornella Russo, Roberto Iacone, Lanfranco D’Elia, Rosalba Giacco, Maria Grazie Volpe, Pasquale Strazzullo

**Affiliations:** 1 Department of Clinical Medicine and Surgery, University of Naples Federico II, 80131 Napoli, Italy; paola.iaccarinoidelson@gmail.com (P.I.I.); ornella.russo@unina.it (O.R.); lanfranco.delia@unina.it (L.D.); strazzul@unina.it (P.S.); 2 Bio Agrifood Department, CNR Institute of Food Science, 83100 Avellino, Italy; rosalba.giacco@isa.cnr.it (R.G.); mariagrazia.volpe@isa.cnr.it (M.G.V.)

**Keywords:** salt reduction, seawater, pizza, hedonic food, water and sodium handling

## Abstract

Seawater is rich in minerals which may help confer good palatability to foods, favouring the use of smaller amounts of salt, a recognized measure of cardiovascular prevention. The aim of this study was to investigate the nutritional properties, sensory characteristics and metabolic effects of a typical Neapolitan pizza prepared with seawater (SWP) in place of common salt, in comparison with Standard traditional Pizza (StP). The nutritional characteristics and the chemical profile of the SWP and StP were assessed by chemical analyses and the use of Food Composition Tables. Twelve healthy volunteers were recruited for a Randomized Controlled Trial, with the consumption of one StP and one SWP using a balanced crossover design. The satiating power and palatability of the two pizzas were tested by the administration of Visual Analogue Scales. Serum glucose, insulin and sodium were measured every 30 min and 3 h urines were collected after each meal. SWP contained nearly 50% less NaCl and a larger amount of micronutrients compared with StP. No significant differences were detected between the two pizzas with regard to satiating power, pleasantness and glycemic and insulinemic response. However, a significant difference was found in the urine volume collected over the 3 h after the two meals (194 mL after StP vs. 292 mL after SWP, *p* = 0.018) and in the 3 h sodium balance (+1.6 g after StP vs. +0.5 g after SWP, *p* = 0.002). Conclusions: SWP appears to be a food with favourable nutritional characteristics, very good acceptability and healthy metabolic properties: these results warrant confirmation by a larger intervention trial.

## 1. Introduction and Aim

Excess dietary salt intake is a recognized causal factor of hypertension and cardiovascular complications [1], nevertheless, salt consumption remains much higher than recommended by health institutions worldwide [2]. The favourable effect of a low sodium diet on blood pressure and cardiovascular disease is supported by several intervention studies [3,4] and food reformulation is part of population strategies aimed at the reduction of salt intake, together with educational campaigns [5]. Bread and bakery products, being the major sources of dietary salt in most countries, are high priority objectives of reformulation policies. The Neapolitan pizza ranks very high in this category due to its large popularity, not only in Italy, being, for example, the top source of sodium among U.S. adolescents [6]. Salt reduction in bakery products may be challenging because NaCl affects the fermentation process and the product shelf life [7], not to mention its impact on tastiness. Multiple approaches have been reported including the use of salt substitutes or flavour enhancers [8], the modification of product structures [9], and different salt distribution and granulometry [10]. Recently, the use of seawater in place of common salt was adopted for the preparation of a type of bread having a reduced content of NaCl while maintaining good palatability and taste compared with traditional bread, together with a higher antioxidant activity [11]. Aside from sodium chloride, seawater contains a rich variety of minerals and small amounts of other substances lending a rich flavour to food, thus making it a suitable potential substitute of common salt [12].

The present article reports the results of a study testing the nutritional properties, the sensory characteristics and the metabolic effects of a novel recipe of Neapolitan pizza featuring the substitution of common salt with commercial seawater in such proportion as to reduce the total amount of sodium chloride in the otherwise traditional dough by approximately 50%.

In preparation for our trial, the SWP sensory properties were preliminarily tested using exploratory triangular tests comparing freshly made SWP and StP prepared by the same chef who later prepared the pizzas for our experiment. Neither professional testers (sensory panel—UNI U590A2520, 2001) nor a group of volunteers, blinded to the type of pizza they were offered, were able to distinguish the SWP from the StP when the pizzas were regularly seasoned with olive oil and tomato sauce (the pizza traditional seasoning).

## 2. Materials and Methods

### 2.1. The Pizzas

The dough for both StP and SWP was prepared from 170 g of organic flour (Agguggiaro and Figna 5 stagioni, 80% type 00/20% type 1, Curatolo, Padova, Italy) with the addition of 110 mL of tap water or commercial seawater respectively. To the StP 0.05 g of brewer’s yeast and 5 g of unrefined salt (Sale Vero, Oro di Sicilia Srl, Nubia di Paceno, Trapani, Italy) were added, while only 0.02 g of yeast and no salt were added to the SWP. Because salt is known to reduce the fermentation rate, based on the calculation that the amount of sodium chloride in SWP would be approximately one half of the StP content, it was decided to use approximately half the amount of yeast while maintaining constant the fermentation time, which was 15 h for both doughs. Commercial seawater was supplied by Steralmar Srl (Bisceglie, BT, Italia).

The 12 StP and the 12 SWP were prepared by the chef with the collaboration of one of the authors (P.I.I) for the implementation of a Randomised Controlled Trial (RCT) on the sensory analysis and the assessment of the satiating power and the metabolic effects of the two pizzas as explained above.

All the pizzas were seasoned with 70 g of organic S. Marzano tomato sauce (Solania, Nocera Inferiore, SA, Italy) with the addition of 0.5 g of salt and 8.5 g of extra virgin olive oil (Azienda Agricola Paragano, Perdifumo, Salerno, Italy), as indicated by the traditional tomato topping recipe of the Pizza chef. Once cooked into the traditional wood oven at the temperature of 450 °C for about 1 min, the pizzas were put in a blast chiller until they hardened in order to preserve their sensory characteristics. Thereafter they were vacuum-sealed and transported in a cooler to be immediately frozen upon arrival to the metabolic kitchen of the Department of Clinical Medicine and Surgery at Federico II University Hospital in Naples, Italy where the test meals were provided a few days later. At the time of consumption, the pizzas were thawed at room temperature and heated for 2 min in a microwave before being served to the trial participants.

### 2.2. Measurement of the Pizzas’ Sodium Chloride Content (the Mohr Method)

The Mohr method uses chromate ions as an indicator in the titration of chloride ions with a silver nitrate standard solution. Forty mL of distilled water were added to 2 g of dried and ground sample (dough or cooked pizza dried in an oven at 105 °C to constant weight); the mixture was stirred for 2 h at room temperature, centrifuged for 10 min at 4000 rpm, filtered in a 50 mL volumetric flask and made up to volume with distilled water. The pH of the solution was adjusted with 0.1 N sodium hydroxide (NaOH) (Merck KGaA, Darmstadt, Germany), up to a value of 8.0. About 2 mL of K_2_CrO_4_ Merck KGaA, Darmstadt, Germany, indicator was added to 20 mL of solution that was titrated with 0.1 N silver nitrate until endpoint.

Parallel, blank tests were carried out by titrating 20 mL of distilled water with 0.1 N silver nitrate until endpoint in the presence of 2 mL of K_2_CrO_4_ indicator.

Based on stoichiometry and number of moles consumed at the endpoint, the amount of chloride was determined. The amount of NaCl Merck KGaA, Darmstadt, Germany, in the sample was derived from the amount of chloride.

### 2.3. Mineralization of the Samples

Acid digestion of pizza samples was performed according to Volpe et al. [13], with slight modifications. To 0.5 g of dried sample, 5 mL of Nitric acid (HNO_3_) Merck KGaA, Darmstadt, Germany, and 2.5 mL of H_2_O_2_, Merck KGaA, Darmstadt, Germany, were added. The mixture was digested at 220 °C until the solution became transparent (Heating digester DK VELP Scientifica). After cooling, the solution was diluted to 25 mL in a volumetric flask. The resulting residue was analysed with Inductively Coupled Plasma-Optical Emission Spectrometry (ICP-OES).

### 2.4. Inductively Coupled Plasma-Optical Emission Spectrometry (ICP-OES) Analysis

The elemental analysis of macro, micro and trace elements was performed by ICP-OES with iCAP 7000 Series (Thermo Scientific, Waltham, MA, USA), equipped with ASX-520 autosampler (CETAC™) according to Barbarisi et al., 2019 [11].

A calibration curve was constructed using standard mix solutions containing the analyzed elements. Working standard solutions were prepared by dilutions of a multi-element (1000 µg mL^−1^) ICP-OES standard (Merck).

A food reference material (NIST 1568b Rice Flour, SIGMA ALDRICH, St. Louis, MO, USA) was digested according to the acid digestion protocol and analysed with ICP-OES, in the same analyses condition of pizza samples.

In order to prevent interference to calibration solutions, 100 µg L^−1^ yttrium solution (Trace Cert, Fluka) was used as internal standard. The element content was calculated by using standard curves and the final concentrations were expressed as mg element kg^−1^ dry weight while for trace elements were expressed as µg element kg^−1^ dry weight.

### 2.5. Volatile Molecules Analysis

The samples were analysed by Solid-Phase Microextraction-Gas Chromatography-Mass Spectrometry (SPME-GC-MS) (Varian, Inc. Walnut Creek, CA, USA), according to the method of Poinot et al. [14], with some modifications.

One gram of crushed pizza was placed in a 20 mL flask with a magnetic stirring bar and 5 µL of 100 ppm 2-methyl-3-heptanone, as the internal standard (Sigma Aldrich, St. Louis, MO, USA). The flask was then sealed with a silicone septum and agitated for 10 s at 500 rpm and then equilibrated at 40 °C for 5 min. Seventy-five mm Carboxen/Polydimethylsiloxane (CAR/PDMS) fiber (Merck KGaA, Darmstadt, Germany) was exposed to the sample head-space shaken with a magnetic stirring bar for 30 min.

At the end of the extraction time, the fibre was inserted into a Varian gas chromatograph model CP 3380, coupled to a Varian Saturn 2000 ion trap mass detector (Varian, Inc. 2700 Mitchell Drive Walnut Creek, CA, USA) for the identification and quantification of volatile compounds.

The flow rates of the helium carrier gas and the oven temperature programming were the following: Initial oven temperature 40 °C, hold time 2 min, Ramp speed 5 °C/min, Final oven temperature 240 °C, Hold time 10 min.

Electron-impact (EI) mass spectra were recorded at ionization energy of 70 eV at a scan rate of 0.9 s/scan, with a scan rate of 3.92 scans/s.

The peak areas of volatile compounds were taken to be their relative abundances and identified by comparing their mass spectra with those contained in the National Institute of Standards and Technology (NIST) database (version 2.0d, Gaithersburg, MD, USA).

### 2.6. RCT Design

The trial was conceived as a double blind balanced randomised crossover trial, where each subject acted as his/her own control by consuming the two different pizzas at one week elapsed time from one another according to a balanced randomised sequence. The trial was approved by the Ethical Committee of the University of Naples Federico II (n. 403.20) and registered at clinicalTrials.gov (NCT04629742).

### 2.7. Participants and Study Protocol

Young and healthy members of the hospital staff were considered eligible for the study on a volunteer basis. Exclusion criteria were smoking, hypertension, diabetes, hyperlipidemia, eating disorders, use of medications affecting satiety and hunger sensations, past gastrointestinal surgery potentially affecting digestion and absorption processes, presence of chronic diseases or strenuous physical activity.

The participants signed an informed consent to participate and received detailed written instructions by a trained researcher about the study protocol: avoid any intense physical activity and complete the evening meal by 9 pm on the day before the test; have a standard breakfast (explained in detail) before 8.30 on the morning of the test; drink at least 300 mL of water between breakfast and the meal test, to guarantee normal hydration, not eating between breakfast and the meal test to guarantee a standard hunger level.

Weight, height and blood pressure (BP) were measured just before the test. A mechanical column scale (Seca model 700, Hamburg, Germany) was used to measure weight to the nearest 0.1 kg, with shoes and heavy clothing removed. Height was measured to the nearest 0.1 cm using the scale integrated stadiometer. Body Mass Index (BMI) (kg/m^2^) was calculated as body weight divided by the square of height. Systolic and diastolic blood pressure and heart rate were measured 3 times with automatic validated devices according to the European Guidelines for the management of arterial hypertension, after having the participant sit for at least 10 min, and the average was recorded. The participants were requested to void just before being weighted and the urines produced thereafter during the 3 h of the test were collected.

The meals were consumed in groups of 4 volunteers at a time under standard conditions in the metabolic kitchen at Federico II University Hospital.

The volunteers were requested to drink 300 mL of water during the meal, which had a maximum duration of 20 min. An additional volume of 300 mL had to be drunk in the 3 h after the meal.

In both meals, at intervals of 30 min for a total of 3 h, a battery of Visual Analogue Scales (VAS) was administered to assess possible differences in the satiating power of the two pizzas. In particular, a VAS, 100 mm in length with words anchored at each end expressing the most positive and the most negative rating, was used to assess hunger, satiety, desire to eat more pizza and prospective food consumption [15,16]. Another similar VAS was used at the end of the meal to evaluate 4 distinctive parameters of sensory analysis (chewiness, flavor, saltiness and general pleasantness). The meaning of each word included in the VAS was explained to the participants before the beginning of the test and repeated on each day of the test.

Blood samples were drawn from an indwelling antecubital venous cannula for the measurement of glucose, insulin and sodium concentration in the fasting state and every 30 min thereafter for 3 h after eating was begun.

Serum cholesterol and triglycerides were measured only in the fasting state. The blood specimens were immediately centrifuged and stored at −70 °C until analyzed. Serum cholesterol, triglyceride, glucose and creatinine levels were measured by an enzymatic colorimetric method (Pentra 400, Horiba ABX, Rome, Italy). Plasma sodium concentration was measured by an ion-selective electrode (ISE) method (Pentra 400, Horiba ABX, Rome, Italy). Serum insulin concentration was measured by ELISA (DIA source, Dusseldorf, Germany).

Plasma osmolarity was calculated by Worthley equation:

Osm = 2 [Na^+^] (mmol/L) + Glucose (mg/dL)/18 + BUN (mg/dL)/2.8 [17].

The volume of the 3 h urine collection was measured, and the sodium and creatinine concentrations were measured by ISE and by an enzymatic colorimetric method.

### 2.8. Statistical Analysis

The statistical analysis was carried out using SPSS for Windows, version 23 (SPSS inc, Chicago, IL, USA). Results were expressed as mean and standard deviation (SD) or standard error of the mean (SEM). To assess the metabolic responses to the two meals, the area under the curve (AUC) of serum glucose, insulin, and sodium concentration measured every 30 min for 3 h and its 95% confidence interval (95% Confidence Interval - CI) was calculated using GraphPad Prism version 8, San Diego, CA, USA [18]. The difference between the two AUC was estimated by unpaired *t*-test by entering the AUC, SD of AUC, and the sample size. Two-sided *p* values < 0.05 were considered statistically significant. The Wilcoxon signed-rank test (matched pairs) was used to analyse the differences in the satiating power and pleasantness of the two pizzas as well as the differences in urine volume and amount of sodium retained after the two meals.

### 2.9. Power Analysis

The sample size was computed by G*Power version 3.1.9.7 (Heinrich Heine University, Düsseldorf, Germany) [19], using the Wilcoxon signed-rank test (matched pairs). At a power of 80% and a significance level of 0.05, a sample size of 12 cross-over study participants provided an effect size of 0.9 concerning the urine volume. As far as the sodium retained, a sample size of 4 participants could have been sufficient to obtain a power of 80% and a significance level of 0.05 (effect size = 2.8). Twelve cross-over study participants provided an actual study power of 100% for the sodium retained. For sensory analysis and satiating power, a 25% variation in individual values was postulated between the StP and SWP pizza. However, sufficient power was achieved only for the sensory variables. The power analysis for carbohydrate metabolism indicated the need for a sample size considerably higher with respect to our pilot study. The effect size was calculated in post hoc analysis as [20,21]: Cohen’s d = difference of means/pooled standard deviations.

## 3. Results

### 3.1. Study Participants

The general characteristics of the trial participants are reported in Table 1. Five women and seven men participated in the study; their age ranged from 21 and 29 years and their mean BMI was 24.8 ± 4.2 kg/m^2^. They were all normotensive and had normal serum cholesterol, triglycerides, glucose, insulin and sodium levels.

### 3.2. Nutritional Characteristics of the Pizzas

Both the StP and the SWP raw pizzas weighed 280 g. They provided 659 kcal and contained 19.3 g proteins, 10.1 g lipids, 130.9 g carbohydrates and 4.5 g fibre. As shown in Table 2, the sodium content of StP exceeded the sodium dietary reference value [22] while SWP did not. Additionally, SWP compared with StP contained much larger amounts of several minerals making a substantial contribution to the achievement of the respective population recommended intakes.

### 3.3. Volatile Profile of the Two Pizzas

The Pizza aroma results from the complex combination of many volatile compounds (VOCs) belonging to different chemical classes, derived from raw ingredients and from their interactions, and originated or modified during dough and cooking processes, including hydration, fermentation, baking, and cooking steps [23].

**Figure 1:**
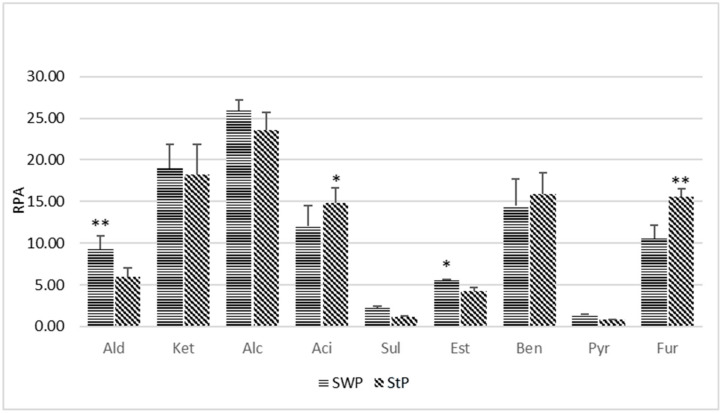
Classes of Volatile Organic Compounds (VOCs) in saltwater pizza (SWP) and standard traditional pizza (StP). Ald = aldehydes; Ket = ketones; Alc = alcohols; Aci = organic acids; Sul = sulphur compounds; Est = esters; Ben Benz = benzenes; Pyr = pyrazines; Fur = furans. Line graphs represent the mean of three experiments (±Standard Deviation). Symbols indicate significance: ∗ *p* < 0.05 with respect to SWP for acid and with respect to StP for esters, ∗∗ *p* < 0.01 with respect to StP for the aldehydes and with respect to furans in SWP. RPA = relative peak area.

In the investigated samples, the SPME-GC-MS) analysis allowed the identification of nine classes of volatile compounds, most of which produced by thermically induced reactions occurring during the cooking process. In Figure 1, the compounds were grouped together by their different prevailing origins. Alcohols were the predominant VOC species (about 26% in SWP and 23.6% in StP, even if the differences are not significant); they do not contribute substantially to the definition of the aroma. Alcohols were followed by ketones (18.3% in StP and 18.9% in SWP) and by benzene derivatives (17.9% in StP and 16.5% in SWP), and both ketones and benzene derivatives showed no significant difference between the two pizza samples. Organic acids (12% in SWP and 14.8% in StP), originating from the oxidation of aldehydes, were significantly more abundant in StP with respect to SWP, while aldehydes (9.3% in SWP and 6.0% in StP), originated from the dissociation of amino acids, showed significance with respect to StP. Esters group (5.6% in SWP and 4.2% in StP) showed a significant difference with respect to StP. Furans were 15.6% in STP and 10.5% in SWP, showing significant differences with respect to SWP. They are responsible for the brown colour and the characteristic smell of cooked culinary products and they naturally form during heated food processing, but they could be dangerous if present in high quantities. Sulfur components (mainly sulphides), which are also formed during the cooking process, were comparable between the two samples (1.3% in SWP and 1.2% in StP). Finally, pyrazines were comparable between the two samples and no significant difference was recorded. They generally contribute to the aroma of pizza (see Supplementary Table S1 for the list of compounds with their relative Chemical Abstracts Service (CAS) numbers and their Relative Peak Areas).

Definitely, SWP contained larger amounts of compounds originated from Maillard reaction and lipid oxidation [24].

### 3.4. Sensory Analysis

The results of the sensory analysis carried out using visual analogue scales are given in Table 3. Although the SWP was perceived as significantly less salty than the StP, this difference did not result in a substantial reduction of its general pleasantness. The differences in chewiness and flavor of the two pizzas were minor and not statistically significant.

**Figure 2:**
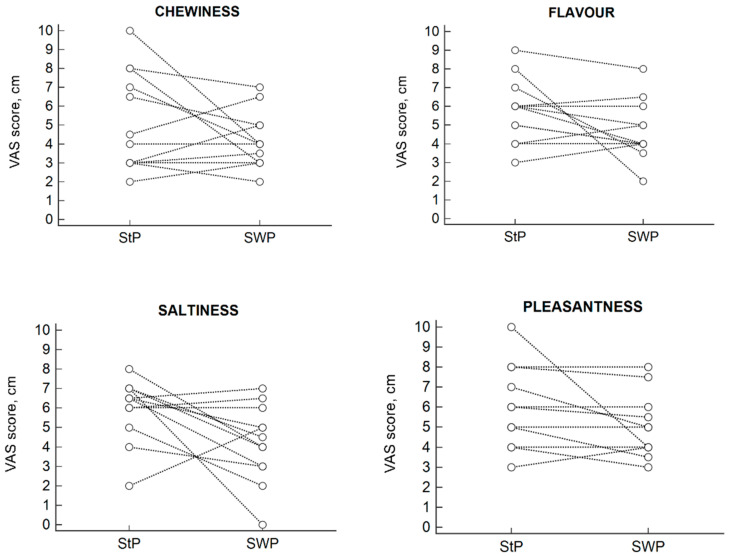
Individual visual analogue scale (VAS) scores for the four factors analysed: chewiness, flavor, saltiness, pleasantness.

Figure 2 shows the individual answers to the VAS for all factors.

### 3.5. Satiating Power

**Figure 3:**
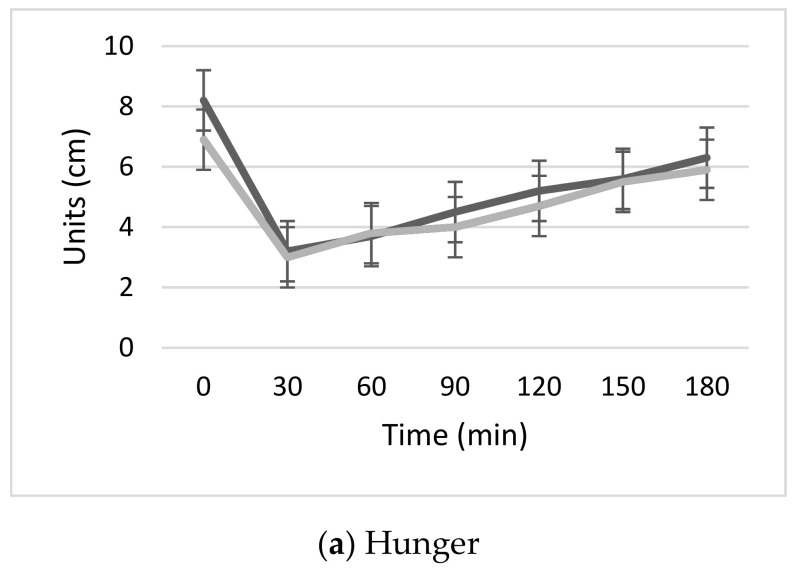

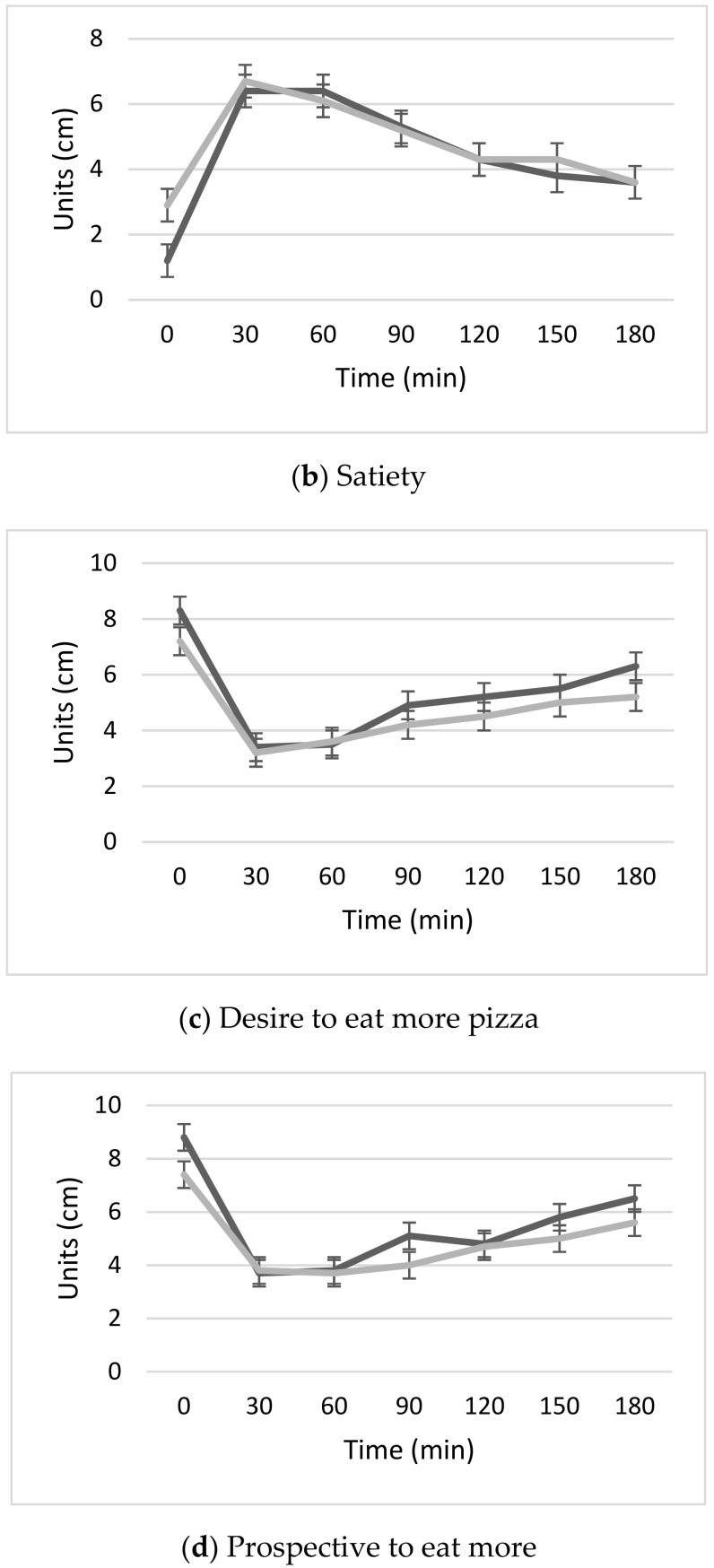
(**a**). Hunger, (**b**). Satiety, (**c**). Desire to eat more pizza and (**d**). Prospective food consumption expressed as mean ± standard error of the mean (SEM) perceived by the participants from the beginning of the test for 3 h, every 30 min, using a 10 cm Visual Analogue Scale (VAS). Dark grey: standard pizza. Light grey: seawater pizza.

Visual analogue scales were also used to assess hunger, satiety, desire to eat more pizza and prospective food consumption before starting the meal test and every 30 min thereafter for 3 h. Figure 3 shows the changes in the same factors after meal consumption on both occasions. The AUC was calculated for all four factors and no significant differences were detected between the two pizzas.

### 3.6. Serum Glucose and Insulin Response

**Figure 4:**
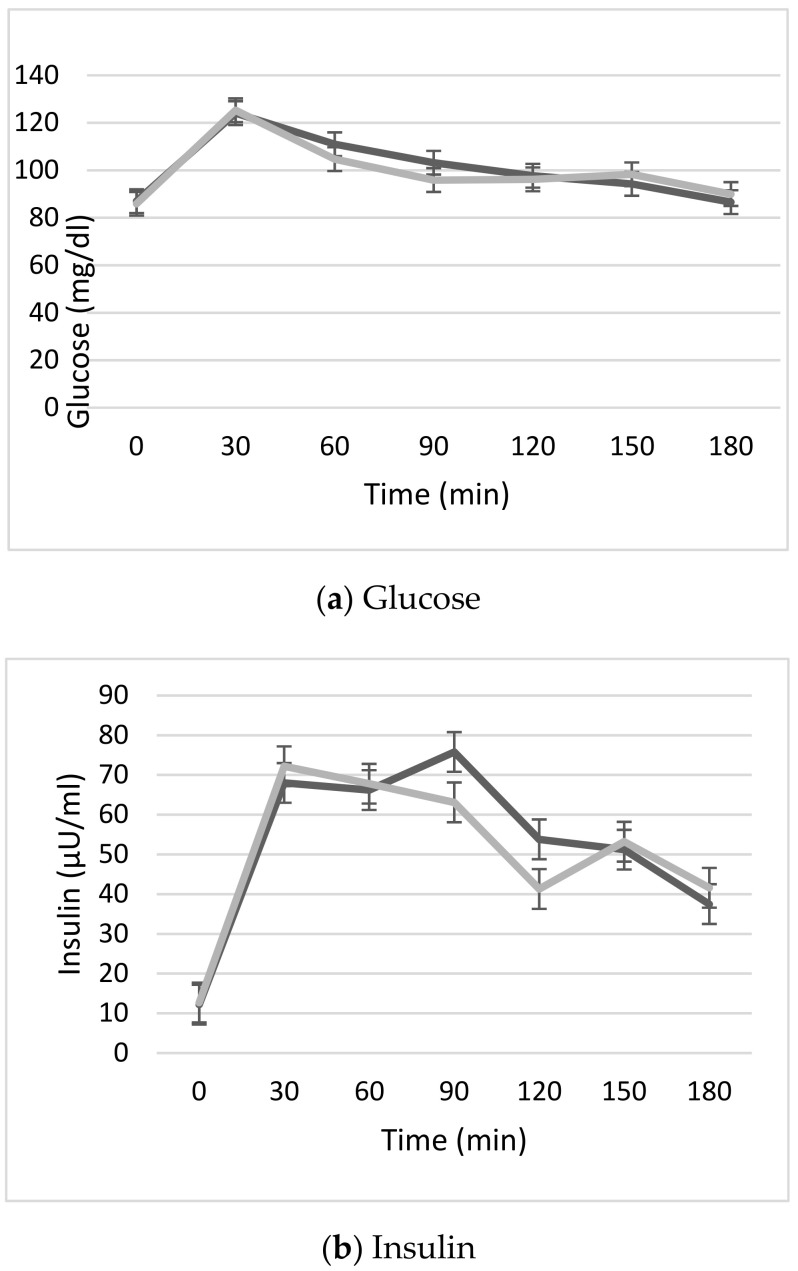
(**a**). Serum glucose (mg/dL) and (**b**). Insulin (µU/mL), expressed as mean ± standard error of the mean (SEM) of the 12 participants from the beginning of the test for 3 h, every 30 min. Dark grey: standard pizza. Light grey: seawater pizza.

The changes observed in serum glucose and insulin levels with the two different meals are shown in Figure 4. No significant differences were found in the AUC for both variables.

### 3.7. Sodium Metabolism

**Figure 5:**
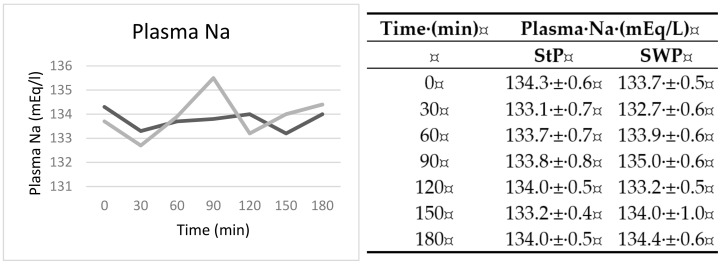
Plasma sodium (mEq/L) expressed as mean ± standard error of the mean (SEM) of the 12 participants from the beginning of the test for 3 h, every 30 min. StP: standard pizza. SWP: seawater pizza. Dark grey: standard pizza. Light grey: seawater pizza.

All participants maintained normal levels of plasma sodium throughout the two tests and no significant differences were observed in the sodium AUC in the two occasions (Figure 5).

**Figure 6:**
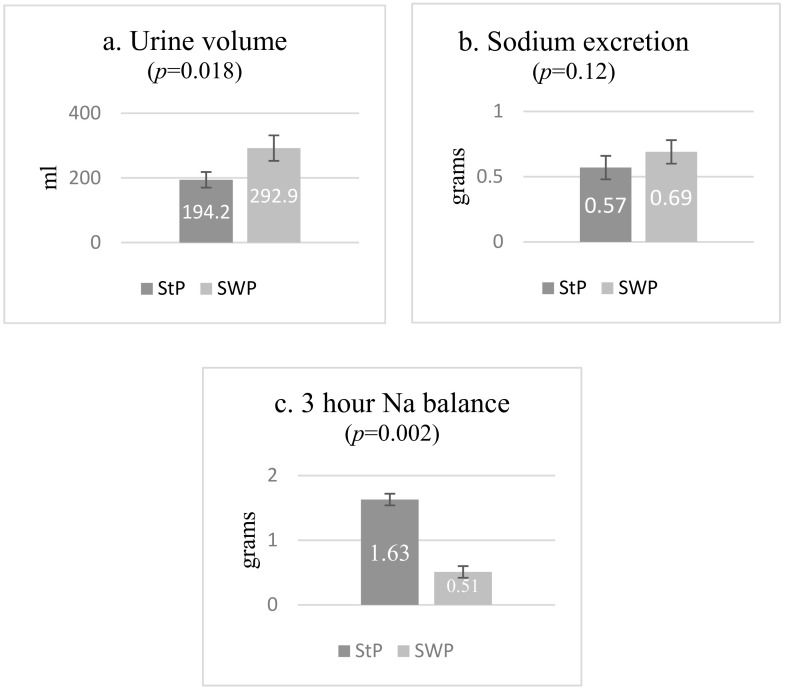
Renal water and Na handling in the 3 h post-meal test. (**a**). Urine volume (mL) collected after 3 h from the consumption of the Standard Pizza (StP) and the Seawater pizza (SWP). *p* = 0.018. (**b**). Urinary Na excretion (g) after the consumption of the Standard Pizza (StP) and the Seawater Pizza (SWP); *p* = 0.12. (**c**). Na retention (g) after the consumption of the Standard Pizza (StP) and the Seawater Pizza (SWP); *p* = 0.002. All results are expressed as mean ± standard error of the mean (SEM).

The results of the water and sodium excretion during the two tests are given in Figure 6. The urine volume produced in the 3 h after the meal was markedly and significantly lower for the standard pizza than for SWP, as reported in Figure 6a.

The amount of sodium excreted over the 3 h of the test was not significantly different in the two occasions (0.57 ± 0.31 g after StP vs. 0.69 ± 0.30 g after SWP, *p* = 0.12), as shown in Figure 6b. These values were 25.9% and 57.5% of the amount of sodium ingested with the respective pizzas. Consequently, as shown in Figure 6c, there was a significant difference in the amount of sodium retained three hours after the meal: 1.6 g (74.1% of the sodium ingested) for StP vs. 0.5 g (42.5% of the sodium ingested) for SWP.

The plasma osmolarity was not different after the two meals.

## 4. Discussion

The main novel findings of the present study were the following:

(1) A 50% lower sodium Neapolitan pizza, made using seawater in place of common salt, was perceived as less salty yet not less tasty, palatable and pleasant as the one made with the traditional recipe. Moreover, no differences were detected in the satiating power.
(2) The SWP provided a biologically significant additional amount of minerals, such as calcium, potassium, magnesium, selenium and iodine, for which the recommended intake is not reached by a large proportion of the paediatric and the adult population.
(3) The SWP had a larger content of some volatile molecules partly produced during the baking process, contributing to the aroma of the pizza.
(4) The consumption of the SWP was associated in the post-prandial period with a significantly larger urine volume and the urinary excretion of a much larger proportion of the sodium ingested compared with StP in face of a similar degree of hydration.
(5) The consumption of SWP and StP provided similar effects on the glycaemic and insulinemic responses to the meal.

To the best of our knowledge, this is the first report of the nutritional features, the sensory and satiety impact and the metabolic responses to a seawater pizza, used as a means to reduce sodium intake. There are other documented attempts to reduce the amount of salt in pizza, e.g., by partially replacing sodium with potassium chloride in the dough [25], differentiating salt granulometry [10], or optimising pizza formulations [7]. In no case, however, was a salt reduction of almost 50% achieved as in our SWP. The good sensory properties and the tastiness acceptability (important driver for food choices [26]) of this new food were likely afforded by the peculiar tastiness of seawater, due to its mineral richness, as shown by the chemical analysis.

In addition to the sensory properties, the SWP had similar satiating power as the StP. Although this result must be considered only a preliminary finding due to the small sample size, it is an encouraging finding; in fact, weak satiation and a higher prospective to eat are among the most influential factors determining a tendency to overconsume, thus, in the long-term, leading to weight gain [15].

Seawater pizza provided large amounts of minerals, which can contribute to the achievement of nutritional requirements of the most important micronutrients. This contribution is all the more important because a widespread tendency to inadequate intake has been consistently observed for all these minerals [27].

The significant differences in renal water and sodium handling observed in the post-prandial period with the consumption of SWP and StP were intriguing on pathophysiological and possibly clinical grounds. The traditional pizza is a highly salty food containing as much as 5.5 g of salt. The excretion of such a large salt load is a challenging task for the kidney and in fact, in our experiment, only approximately one-fourth of the load was excreted in the 3 h post-prandial period, despite that the subjects were kept well hydrated throughout the test. The fact that the absolute amount of sodium excreted was similar to the two types of pizza in spite of the 50% difference in their respective sodium content suggests that the rate of sodium excretion was near its maximum capacity. As a consequence, there was a three-fold larger amount of sodium retained at the end of the 3 h post-prandial period, to be eliminated later, presumably over many hours, in order to re-establish a neutral sodium balance. In parallel, there was larger water retention with the StP as only one-third of the water load given during the test was eliminated compared with about one half with SWP.

It is possible that the different mineral content of the two pizzas may have played a role in the respective rates of sodium and water excretion by affecting the secretion of glucagon, ADH or aldosterone, but we have no evidence in this respect.

A delay in the excretion of a sodium load is a well-documented phenomenon [28]. Bankir et al. reported in diabetic patients that a lower rate of sodium and water excretion during daytime was followed by a compensatory rise during the night, at the cost however of higher blood pressure and a lower nocturnal blood pressure fall [29]. It is not known if similar alterations may occur as a consequence of a particularly significant albeit occasional salt load also in susceptible though clinically healthy subjects.

Finally, the similarities in the glucose and insulin responses to the two different meals detected in our study are in accordance with the conclusions of a systematic review, which analysed the available intervention trials assessing the effect of sodium reduction on glucose tolerance [30] and highlighted the controversial results of the different trials.

The major strengths of our study are: (1) the comprehensive analysis of the different aspects relevant to the nutritional characterization of this novel lower sodium food (nutritional composition, sensory and satiety analysis and short term metabolic effects); (2) the randomised control design; (3) the carefully standardized experimental conditions and the continuous monitoring of satiation factors, glucose and insulin responses and renal sodium and water handling.

The limitations are given by the relatively small sample size of our RCT for the analysis of the glycaemic and insulinemic response and the evaluation of the satiation power, and by the absence of a more prolonged period of urine collection, which could have provided additional information on the renal water and sodium handling after the pizza salt load.

## 5. Conclusions and Perspectives

In summary, we showed that the seawater pizza tested in our study in comparison with the traditional pizza recipe, while allowing a 50% reduction in salt intake, was not inferior with regard to tastiness and level of satiation to the traditional one: in addition, it was associated with the provision of several minerals often underrepresented in the population habitual diet and with a faster excretion of the sodium load provided by pizza.

Although our results warrant confirmation by a larger intervention trial, they are encouraging about the potential use of seawater for the domestic as well as industrial preparation of healthy foods functional to the reduction of sodium intake. Food reformulation is one of the pillars of any strategy of dietary salt reduction at the population level and the use of ingredients capable to improve tastiness in spite of the reduction of salt content is a major tool for both industrial and home food preparation. The provision of essential minerals other than sodium is an additional advantage of the use of seawater in place of common salt. Further studies are needed to fully exploit the potential of seawater as a salt substitute for food reformulation.

## Figures and Tables

**Table 1:** General characteristics of the study participants.

*N* = 12 (5F, 7M)	Min	Max	Mean ± SD
BMI (kg/m^2^)	16.1	30.9	24.8 ± 4.2
P/A (SBP/DBP) (mmHg)	102/59	137/81	117/74 ± 10/7
COL tot (mg/dL)	128.0	217.0	163.5 ± 27.5
HDL (mg/dL)	38.8	61.7	53.0 ± 7.1
LDL (mg/dL)	63.0	143.0	97.4 ± 23.8
TG (mg/dL)	39.0	110.0	65.25 ± 21.7
GLU t’0 (mg/dL)	81.0	94.0	87.0 ± 4.0
INS t’0 (µU/mL)	5.8	22.3	12.2 ± 5.5
Na t’0 (mMol/L)	130.1	136.9	134.3 ± 2.0

SD: standard deviation; BMI: body mass index; P/A: blood pressure; SBP: systolic blood pressure; DBP: diastolic blood pressure; COL tot: total cholesterol, HDL: HDL cholesterol; LDL: LDL cholesterol; TG: triglycerides; GLU t’0: serum glucose before meal test, INS t’0: serum insulin before meal test; Na t’0: serum sodium before meal test.

**Table 2:** Nutritional characteristics of the pizzas.

Micronutrient	StP	% of Recommended AI or PRI StP	SWP	% of Recommended AI or PRI SWP
Na (g)	2.20	130% for people <60 years old 160% for people >60 years old	1.20	80% for people <60 years old 100% for people >60 years old
Ca (mg)	58.61	6%	105.50	10%
K (mg)	226.80	6%	390.88	10%
Mg (mg)	58.56	24%	214.90	90%
Fe (mg)	3.03	17% (women) 30% (men)	5.66	31% (women) 56.5% (men)
I (µg)	2.80	2%	11.20	7.5%
Zn (mg)	1.18	13% (women) 10% (men)	2.15	24% (women) 18% for (men)
Se (mg)	0.01	18%	0.17	309%

StP: standard pizza. SWP: seawater pizza. Na: sodium. Ca: calcium. K: potassium. Mg: magnesium. Fe: iron. I: iodine. Zn: zinc. Se: selenium. PRI: population reference intake. AI: adequate intake.

**Table 3:** Difference in participants’ perception of chewiness, flavor, saltiness and general pleasantness between the two pizzas at the end of the meal.

	StP (Mean ± SEM)	SWP (Mean ± SEM)	*p*
Chewness	5.2 ± 0.8	4.2 ± 0.4	0.28
Flavour	5.8 ± 0.5	4.7 ± 0.5	0.07
Saltiness	5.9 ± 0.5	4.5 ± 0.5	0.04
General pleasantness	5.9 ± 0.6	5.0 ± 0.4	0.08

StP: standard pizza. SWP: seawater pizza. Results are expressed as mean ± standard error of the mean (SEM).

## Supplementary Materials

The following are available online at https://www.mdpi.com/2072-6643/12/11/3533/s1, Table S1: Volatile organic compounds identified in sea water pizza(SWP) vs. Standard pizza (StP).
