# Supplementary material for: A Lower Sodium Neapolitan Pizza Prepared with Seawater in Place of Salt: Nutritional Properties, Sensory Characteristics, and Metabolic Effects
Source: Nutrients. 2020 Nov 17;12(11):3533. doi: 10.3390/nu12113533 (PMC7698524; doi:10.3390/nu12113533)
Supplement: Supplementary file 1 [file nutrients-12-03533-s001.pdf]

SUPPLEMENTARY TABLE

Supplementary Table Volatile organic compounds identified in sea water pizza(SWP) vs. Standard pizza (StP). The data are expressed by Relative Peak Area (Area peak compound/Area peak Internal Standard x 100). Mean values obtained by triplicate determinations on two distinct pizzamaking replicate

Compounds	SwP	StP
2-Methylpropanal(CAS 78-84-2)	5.72±1.51	3.76±0.06
2-Methylbutanal(CAS 96-17-3)	1.38±0.78	0.55±0.02
2-Heptenal(CAS 18829-55-5)	1.38±0.76	0.55±0.02
2-Methylfuran(CAS 534-22-5)	1.18±0.06	3.27±0.64
Furfural(CAS 98-01-1)	6.1±1.07	7.42±0.88
2-Acetylfuran (CAS 1192-62-7)	2.29±0.04	4.14±0.31
5-Methylfurfural (CAS 620-02-0)	0.11±0.01	0.76±0.08
2,3-Pentanedione(CAS 600-14-6)	1.85±0.06	3.89±0.37
3-Hydroxy-2-butanone (CAS 51555-24-9)	15.83±2.71	12.75±1.91
2-Octanone(CAS 111-13-7)	0.96±0.4	0.58±0.04
3-Methyl-butanol(CAS123-51-3)	5.84±1.86	2.78±0.09
2-Phenylethanol(CAS60-12-8)	12.43±2.5	16.69±1.06
1-Octen-3-ol(CAS3391-86-4)	4.58±0.97	5.56±1.62
2,5-Dimethylpyrazine(CAS123-32-0)	0.23±0.04	0.19±0.01
Methylpyrazine(CAS109-08-0)	0.71±0.05	0.54±0.03
Ethylpyrazine(CAS13925-00-3)	0.35±0.07	0.14±0.01
1,2-Dimethylbenzene(CAS95-47-6)	0.67±0.07	2.16±0.67
Benzadehyde(CAS100-52-7)	9.54±1.63	10.83±3.46
Dimethylsulphide(CAS75-18-3)	1.73±0.06	0.76±0.08
Dimethyltrisulfide(CAS3658-80-8)	0.46±0.02	0.65±0.05
Hexanoic acid(CAS 142-62-1)	5.03±0.76	5.31±0.54
Decanoic acid(CAS334-48-5)	6.65±1.31	9.11±0.25
Ethyloctanoate(CAS106-32-1)	3.20±0.96	2.86±0.73
